# The Effect of a High-Protein Diet Supplemented with Blackthorn Flower Extract on Polyphenol Bioavailability and Antioxidant Status in the Organs of C57BL/6 Mice

**DOI:** 10.3390/nu15184066

**Published:** 2023-09-20

**Authors:** Vedran Balta, Domagoj Đikić, Irena Landeka Jurčević, Dyana Odeh, Nada Oršolić, Nikola Ferara, Dario Dilber, Petar Dragičević, Verica Dragović-Uzelac

**Affiliations:** 1 Faculty of Science, University of Zagreb, Rooseveltov Trg 6, 10000 Zagreb, Croatia; domagoj.djikic@biol.pmf.hr (D.Đ.); dyana.odeh@biol.pmf.hr (D.O.); nada.orsolic@biol.pmf.hr (N.O.); 2 Faculty of Food Technology and Biotechnology, University of Zagreb, Pierottijeva 6, 10000 Zagreb, Croatia; ilandeka@pbf.hr (I.L.J.); vdragov@pbf.hr (V.D.-U.); 3 Department of Dermatovenereology, University Hospital Centre Sestre Milosrdnice, Vinogradska Cesta 29, 10000 Zagreb, Croatia; nfera94@gmail.com; 4 Magdalena Clinic for Cardiovascular Diseases, Ljudevita Gaja 2, 49217 Krapinske Toplice, Croatia; dario.dilber@gmail.com; 5 University Hospital Centre Zagreb, 12 Kišpatićeva St, 10000 Zagreb, Croatia; pd4082@gmail.com

**Keywords:** *Prunus spinosa* L., polyphenol compounds, biodistribution, bioactivity, interaction polyphenols with whey proteins

## Abstract

The health benefits of polyphenols are based on their bioavailability, which is why a significant portion of research focuses on factors that affect their bioavailability. Previous studies suggest that the intake of polyphenols along with macronutrients in food represents one of the key factors influencing the bioavailability of polyphenols and, consequently, their biological activity in the organism. Since polyphenols in the human diet are mainly consumed in food together with macronutrients, this study investigated the in vivo absorption, metabolism, and distribution of polyphenolic compounds from the water extract of blackthorn flower (*Prunus spinosa* L.) in combination with a protein-enriched diet in the organs (small intestine, liver, kidney) of C57BL/6 mice. The bioaccumulation of polyphenol molecules, biologically available maximum concentrations of individual groups of polyphenol molecules, and their effect on the oxidative/antioxidative status of organs were also examined. The results of this study indicate increased bioabsorption and bioavailability of flavan-3-ols (EC, EGCG) and reduced absorption kinetics of certain polyphenols from the groups of flavonols, flavones, and phenolic acids in the organs of C57BL/6 mice after intragastric administration of the water extract of blackthorn flower (*Prunus spinosa* L.) in combination with a diet enriched with whey proteins. Furthermore, subchronic intake of polyphenols from the water extract of blackthorn flower (*Prunus spinosa* L.) in combination with a diet enriched with whey proteins induces the synthesis of total glutathione (tGSH) in the liver and superoxide dismutase (SOD) in the liver and small intestine. The results of this study suggest potential applications in the development of functional foods aimed at achieving the optimal health status of the organism and the possibility of reducing the risk of oxidative stress-related disease.

## 1. Introduction

Blackthorn (*Prunus spinosa* L.) is a perennial shrub belonging to the rose family (*Rosaceae*), growing throughout Europe, western Asia, and north-western Africa [1,2]. Due to extremely interesting medicinal properties and pharmacological actions, in recent years, the industrial and pharmacological properties of all parts of the blackthorn plant are being tested as a potential source of polyphenols, nutrients, and functional foods [3,4,5,6,7]. All the parts of blackthorn, especially the flowers, contain high masses of polyphenols such as quercetin, campherol, and rutin and represent a suitable source of a mixture of polyphenols for testing the affinity of their intestinal bioabsorption and thus their potential bioactive effects in vivo [8,9,10]. Since the blackthorn flowers and fruits contain a number of antioxidant polyphenolic compounds which have bioactive properties in human and animal organisms, they are used as traditional medicinal preparations in folk medicine in the treatment of gastrointestinal, respiratory, cardiovascular, urinary and kidney problems, hypertension, hypercholesterolemia, and inflammatory responses in an organism [11,12,13,14]. Furthermore, studies by Marchelak et al. [15] have proven that the in vivo application of the blackthorn flower in humans is effective in increasing the antioxidant capacity and reducing the concentration of 3-nitrothyrosine and lipid peroxides in plasma whose damage is caused by peroxynitrite. Also, the recent in vitro antitumor tests with flower, fruit, and drupe extract have yielded promising results in the chemoprevention of tumor diseases [6,16,17,18]. In addition to the above-mentioned, the results of our recent study show that the polyphenols of the blackthorn flower aqueous extract and their metabolites in the in vivo mouse model C57BL/6 after a daily subchronic oral administration at a dose of 25 mg/kg body weight for a duration of 28 days reduce the oxidative stress and activate enzymes involved in the antioxidant defense system [6].

Since the health benefits of polyphenols are based on their bioavailability, much of the research focuses on the factors that affect their bioavailability [19]. Previous research suggests that the intake of polyphenols together with food macronutrients (carbohydrates, proteins, lipids) is one of the key factors influencing the bioavailability of polyphenols and, therefore, influencing their biological activity in the organism. However, most of these studies are focused on the interactions of polyphenols and macronutrients in in vitro conditions, and a relatively small number of studies describe their bioavailability and bioactivity in combination with macronutrients in an in vivo model [20,21,22,23]. Since, in the human diet, polyphenols are mainly consumed in food along with macronutrients, based on the results of our recent research, in this research, we investigated the bioavailability of the polyphenols of the blackthorn flower aqueous extract in combination with a diet enriched with proteins and their influence on the oxidation/antioxidation status in mouse organs C57BL/6.

## 2. Materials and Methods

### 2.1. Blackthorn Flower Aqueous Extract and Whey Proteins

The blackthorn flower samples were obtained from the manufacturer Suban Ltd. company (Samobor, Croatia), a certified collector and producer of medicinal plants, and they were part of batch number 63451. The preparation of blackthorn flower aqueous extract and determination of the quantitative and qualitative content of total polyphenols are detailed in the works of Elez Garofulić et al. [1] (see Tables 1 and 2) and Lovrić et al. [24].

As a source of a high-protein diet, whey protein from the manufacturer Myprotein (Berlin, Germany) was used, prepared as a 10% aqueous solution.

### 2.2. Animals

Male C57BL/6 mice, 3 months old, obtained from the Department of Animal Physiology, Faculty of Science, were used in the study. The study was performed on a total of 120 C57BL/6 mice divided into 4 groups of *n* = 6 animals, each according to the treatment. Animals were fed with a standard diet for laboratory animals (Standard Diet 4RF21 GLP certificate, Mucedola, Settimo Milanese, MI, Italy) described in work by Đikić et al. [10] and Balta et al. [9]. The availability of food and water was *ad libitum*, and housing conditions were standard (daily rhythm of 12 h and 12 h of darkness, temperature 24 °C with controlled humidity).

The research was approved by the Ethical Committee of the Faculty of Science, University of Zagreb (approval code: 251-58-10617-14-21) and was performed in accordance with ethical and legal principles valid in the Republic of Croatia (Law on Animal Welfare, NN 102/2017 [25]; Law on Amendments to the Law on Animal Welfare, NN 37/13 [26]; Regulation on the Protection of Animals Used for Scientific Purposes, NN 55/13 [27]), and according to the Guide for the Care and Use of Laboratory Animals, DHHS (NIH), Publ # 86-23, National Research Council [28].

### 2.3. Experimental Design and Doses

Mice were individually labeled and weighed (digital scales ABS 220-4, Kern Sohn, Balingen, Germany) before the start of the experiment, as well as during the experiment, and were thereby classified into groups with approximately similar body weight (weight 25 ± 2 g). Based on the mean body weight of the animals per cage for each group, the number of individual preparations given during the experiment was determined.

Animals were weighed at the beginning of the experiment, every day during the experiment, and on the day of the sacrifice. During the continuous experiment, the animals were orally administered with various preparations via intragastric (*ig*) route every day for 28 days using a gastric cannula. The groups and treatment methods for the animals are the following:

Cont.—Control group (0.3 mL of physiological saline);

EPS—Blackthorn flower extract group (0.3 mL, 25 mg/kg of body weight per day);

WP—Whey protein group (0.2 mL, 700 mg/kg of body weight per day);

EPS + WP—Hawthorn flower extract group (0.3 mL, 25 mg/kg of body weight per day) + whey protein (0.2 mL, 700 mg/kg of body weight per day).

The animals were sacrificed on the 1st, 7th, 14th, 21st, and 28th days of the experiment. During the sacrifice procedure, all the animals were adequately anesthetized and analgesized by intraperitoneal (*ip*) administration of a combination of Narketan^®^ Vetoquinol S.A., BP 189 Lure Cedex, France (active substance Ketamine), and Xylapan^®^ Vetoquinol Biowet Sp., Gorzow, R. Poland (active substance Xylazine), at a dose of 25 mg/kg body weight.

### 2.4. Determination of the Bioavailability of the Blackthorn Flower Aqueous Extract Polyphenols in Mouse Organs

The samples for analyzing the concentration of polyphenolic compounds in the organs of mice (specifically, the small intestine, liver, and kidney) underwent testing across all experimental groups on 1st, 7th, 14th, 21st, and 28th days of the experiment, by using high-performance liquid chromatography (UPLC) with MS/MS detection, as recommended by Ganguly et al. [29]. The sample preparation for enzymatic hydrolysis of the organs, enzymatic hydrolysis, tissue sample extraction, and determination of phenolic compounds in the tissues were thoroughly described in the works of Đikić et al. [10] and Balta et al. [9].

### 2.5. Markers of Oxidative Stress

Small intestine, liver, and kidney were used to measure markers of oxidative stress and were prepared as described in our previous paper [9,10]. The analyses of malondialdehyde (MDA), superoxide dismutase (SOD), catalase (CAT), and total glutathione (tGSH) were conducted with homogenate supernatants by methods of the antioxidative defense system, previously described in detail in Landeka et al. [30], and here it is described only briefly. Protein concentration in tissue samples was used to express the values of the measured oxidative stress parameters with bovine serum albumin (BSA) as the standard [31].

Lipid peroxidation was determined by measuring the concentration of MDA using a modified method of Ohkawa et al. [32] for the detection of thiobarbiturate reactive species (TBARS) whose concentration is measured spectrophotometrically (Libra S22, Biochrom, Cambridge, UK) at a wavelength of 532 nm. The MDA levels were determined using the molar absorption coefficient for the malondialdehyde–thiobarbiturate (MDA–thiobarbituric acid, TBA) complex of 1.56 × 10^5^ M^−1^ cm^−1^.

Catalase (CAT) activity assay in tissues was assayed by measuring the initial rate of H_2_O_2_ disappearance at 240 nm with Libro S22 spectrophotometer (Biochrom) [33]. Catalase activity was calculated using the extinction coefficient of H_2_O_2_ (ε = 39.4 mM^−1^ cm^−1^).

The superoxide dismutase (SOD) assay is a modification of the method by Flohé and Ötting [34]. The SOD activity was calculated from the percentage of inhibition of the reaction of xanthine oxidation (Δ*A*/min ≈ 0.025), which creates a superoxide anion as a substrate for the SOD present in the samples. The superoxide anion not used by the enzyme oxidizes the cytochrome. Regarding total glutathione, in brief, 20 μL of sample, 40 μL of 0.035 M HCl, and 40 μL of 10 mM 5–5′-dithiobis-2-nitrobenzoic acid (DTNB, Ellman’s Reagent) was added to each microplate well. The absorbance was measured at 412 nm (Microplate reader Model 550, Bio-Rad, Hercules, CA, USA). One unit of total SOD activity was defined as the amount of enzyme required to achieve 50% inhibition in the typical calibration curve obtained with standard SOD type I. Horse heart cytochrome C (type VI), human blood SOD (type I, lyophilized powder, 2400 U/mg protein), xanthine, and xanthine oxidase (200 U/mL) were from Sigma-Aldrich, St. Louis, MO, USA.

The total glutathione (tGSH) assay is a modification of the method first described by Tietze [35] and in the work of Landeka Jurčević et al. [30]. In a 96-well plate, 40 μL of 10 mM 5-5′-dithiobis (2-nitrobenzoic acid) (DTNB, Ellman’s Reagent) was mixed with 20 μL of sample supernatant, pre-treated with 40 µL of 0.035 M HCL, incubated for 10 min, and measured at 412 nm in an ELISA plate reader (Biorad Laboratories, Hercules, CA, USA). Then, the 100 µL of the reaction solution contained the following: 9980 µL of 0.8 mM NADPH and 20 µL of glutathione reductase. A total of 0.2 U/mL was added to the ELISA plate reader, and the absorbance was read at 412 nm every minute for 5 min. GSH levels were determined using molar absorption coefficients for DTNB of 8.22 M^−1^ cm^−1^. DTNB and NADPH, glutathione reductases were purchased from Sigma, St. Louis, MO, USA.

### 2.6. Statistical Analysis

The data were presented as mean ± standard deviation (SEM ± SD). Statistical analysis was performed using the STATISTICA 14 program (StatSoft, Tulsa, OK, USA) [36], and GraphPad Prism v. 17.0 [37] was used for visualization of data Using the Kruskal–Wallis nonparametric test, the data were analyzed and considered significant at the level of *p* ≤ 0.05. A further analysis of the differences between the groups was made by multiple comparisons of the mean values of all groups.

## 3. Results

The bioaccumulated concentrations of detected polyphenolic molecules in the small intestine, liver, and kidneys can be categorized and described as high (in the range of 4.5–5.5 µg/g tissue), relatively high (in the range of 3.5–4.5 µg/g tissue), moderate (in the range of 1.5–3.5 µg/g tissue), low (0.5–1.5 µg/g tissue), and very low (0–0.5 µg/g tissue) concentrations. Below are the results of the bioaccumulation of polyphenols in the mentioned organs.

### 3.1. Bioaccumulation of Polyphenols in the Small Intestine after 28 Days of Treatment with Extract P. spinosa with a Whey-Protein-Enriched Diet

**Figure 1:**
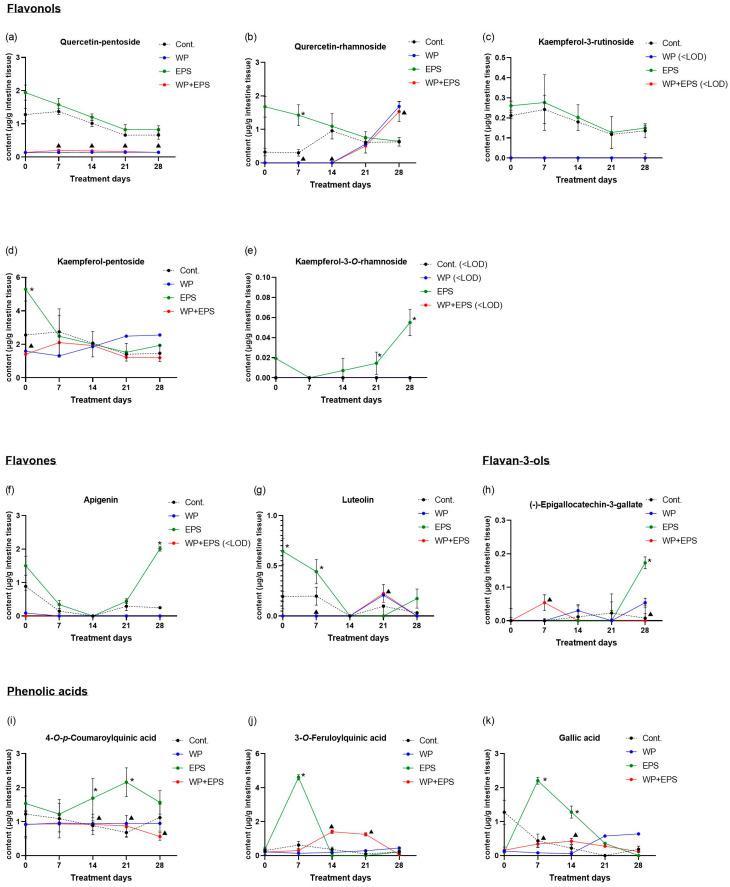
Pharmacokinetic curves of polyphenol bioavailability in the small intestine of C57BL/6 mice after intragastric administration of hawthorn flower extract (25 mg/kg of body weight) and whey proteins (700 mg/kg of body weight) once daily for 28 days. The graphs depict polyphenolic compounds whose pharmacokinetic parameters (*c*_max_/AUC_last_) are statistically significantly different (*p* ≤ 0.05) between the EPS and EPS + WP groups. There were six animals per group (*n* = 6). * Statistically significant compared to the control group (*p* ≤ 0.05); ^▲^ statistically significant difference compared to the EPS group (*p* ≤ 0.05). The results are presented as the mean ± standard deviation. Abbreviations: Cont.—control; EPS—hawthorn flower water extract; WP—whey proteins; <LOD—below the limit of detection.

**Figure 2:**
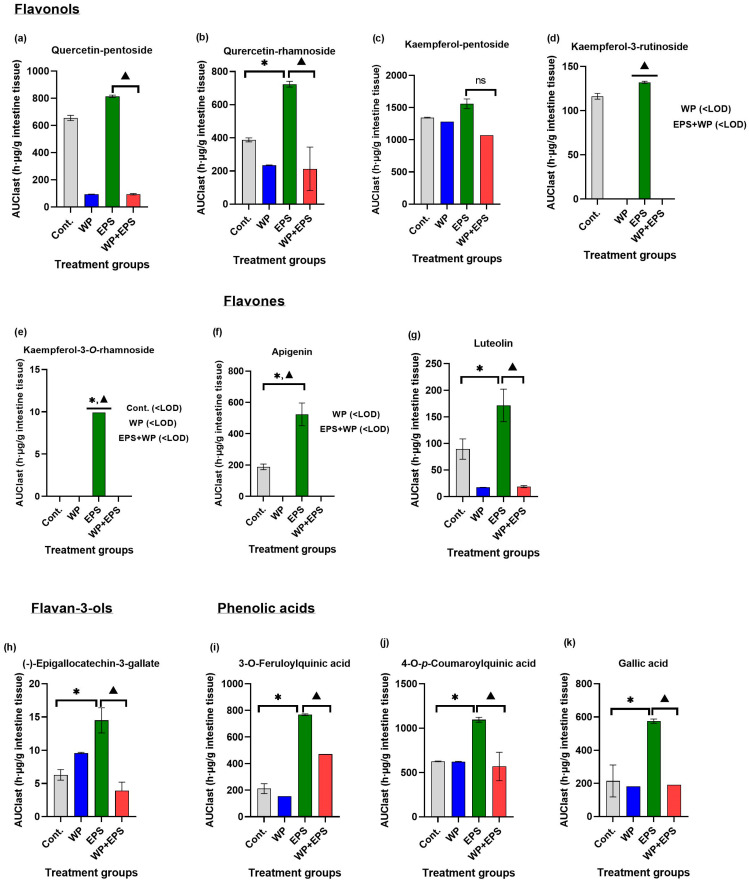
The area under the curve (AUC) values of polyphenols bioavailability in the small intestine after intragastric administration of blackthorn flower extract (25 mg/kg of body weight) and whey proteins (700 mg/kg of body weight) once daily for 28 days. The graphs depict polyphenolic compounds whose pharmacokinetic parameters (*c*_max_/AUC_last_) are statistically significantly different (*p* ≤ 0.05) between the EPS and EPS + WP groups. There were six animals per group (*n* = 6). * Statistically significant compared to the control group (*p* ≤ 0.05); ^▲^ Statistically significant difference compared to the EPS group (*p* ≤ 0.05). The results are presented as the mean ± standard deviation. Abbreviations: Cont.—control; EPS—hawthorn flower water extract; WP—whey proteins; <LOD—below the limit of detection; ns—not statistically significant.

After subchronic dosing of experimental animals with blackthorn flower extract and whey proteins, a total of 25 polyphenolic compounds were detected in the small intestine, constituting 78.12% of the total number of polyphenolic compounds found in the water extract of blackthorn flowers. A statistically significant difference in *c*_max_ or AUC_last_ values (*p* ≤ 0.05) in the small intestine was observed for 11 polyphenolic compounds between the groups treated with blackthorn flower extract (EPS) and blackthorn flower extract in combination with a protein-enriched diet (EPS + WP), accounting for 34.37% of the total compounds found in the blackthorn flower extract and 44.0% of the total compounds detected in the small intestine (Table 1, Figure 1 and Figure 2).

Among flavonol compounds, the highest bioabsorption throughout the entire treatment period in the EPS and EPS + WP groups, with no statistically significant difference (*p* ≤ 0.05), was observed for quercetin-3-*O*-rutinoside. Moderate concentrations were determined in both groups for quercetin-rhamnoside, with concentrations of quercetin-rhamnoside in the EPS + WP group reaching zero by the 14th day of treatment, after which its concentration had been increasing up to the 28th day of the treatment, while during the same time frame, the concentration of quercetin-rhamnoside decreased in the EPS group (Figure 1b). The greatest difference in concentration between the EPS and EPS + WP groups was found for quercetin-pentoside, with its concentration in the EPS + WP group remaining very low throughout the entire duration of the experiment (Figure 1a). Bioabsorption of other quercetin compounds in the EPS and EPS + WP groups was similar, with their concentrations in the relatively low concentration range. Among kaempferol compounds, the highest concentration and the greatest difference in concentration, which remained high in the EPS group and moderate in the EPS + WP group, were found for kampferol-pentoside up to the 14th day of treatment (Figure 1d). The bioabsorption of other kaempferol metabolites between the EPS and EPS + WP groups was similar and remained in the low and very low concentration range.

Analyzing the biodynamics of the flavones apigenin and luteolin between the EPS + WP and EPS groups, apigenin concentration in the small intestine in the EPS + WP group remained at zero throughout the entire treatment, while luteolin reached its highest value, which remained in the very low concentration range in the EPS + P group, on the 21st day of treatment compared to the EPS group (Figure 1f,g). The concentration values of catechin group molecules in the small intestine remained in a very low concentration range, both in the EPS group and in the EPS + WP group (Figure 1h). The biodynamics of phenolic acids between the EPS and EPS + WP groups were similar, with smaller maximum concentrations and extended time to reach their maximum concentrations observed in the EPS + WP group for 3-*O*-feruloylquinic, 4-*O*-*p*-coumaroylquinic, and gallic acid (Figure 1i,j,k)

### 3.2. Bioaccumulation of Polyphenols in the Liver after 28 Days of Treatment with Extract P. spinosa with a Whey-Protein-Enriched Diet

**Figure 3:**
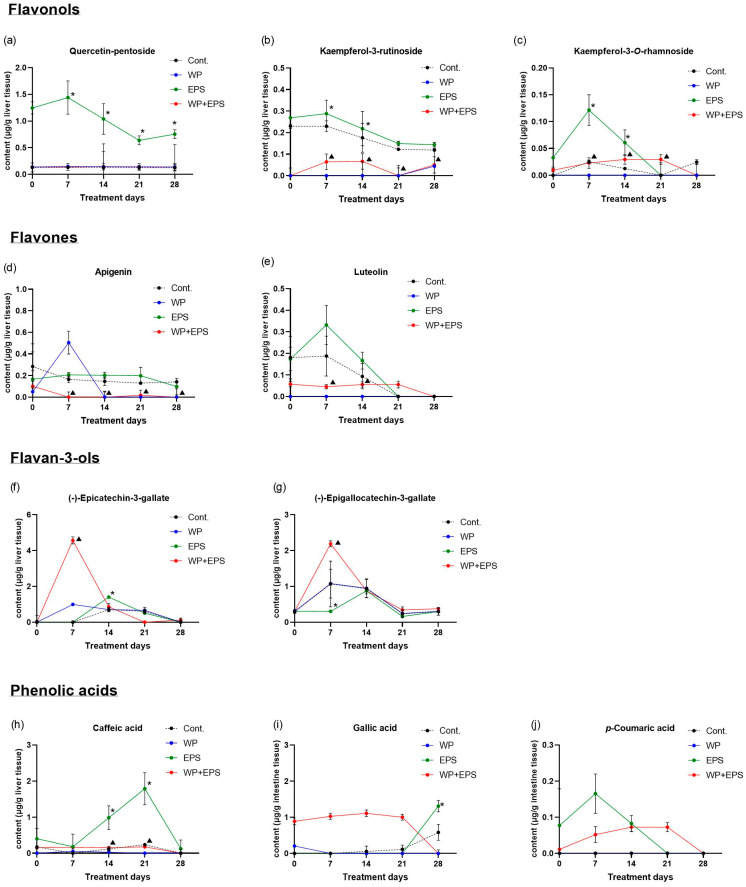
Pharmacokinetic curves of polyphenol bioavailability in the liver of C57BL/6 mice after intragastric administration of blackthorn flower extract (25 mg/kg of body weight) and whey proteins (700 mg/kg of body weight) once daily for 28 days. The graphs depict polyphenolic compounds whose pharmacokinetic parameters (*c*_max_/AUC_last_) are statistically significantly different (*p* ≤ 0.05) between the EPS and EPS + WP groups. There were six animals per group (*n* = 6). * Statistically significant compared to the control group (*p* ≤ 0.05); ^▲^ Statistically significant difference compared to the EPS group (*p* ≤ 0.05). The results are presented as the mean ± standard deviation. Abbreviations: Cont.—control; EPS—hawthorn flower water extract; WP—whey proteins; <LOD—below the limit of detection.

**Figure 4:**
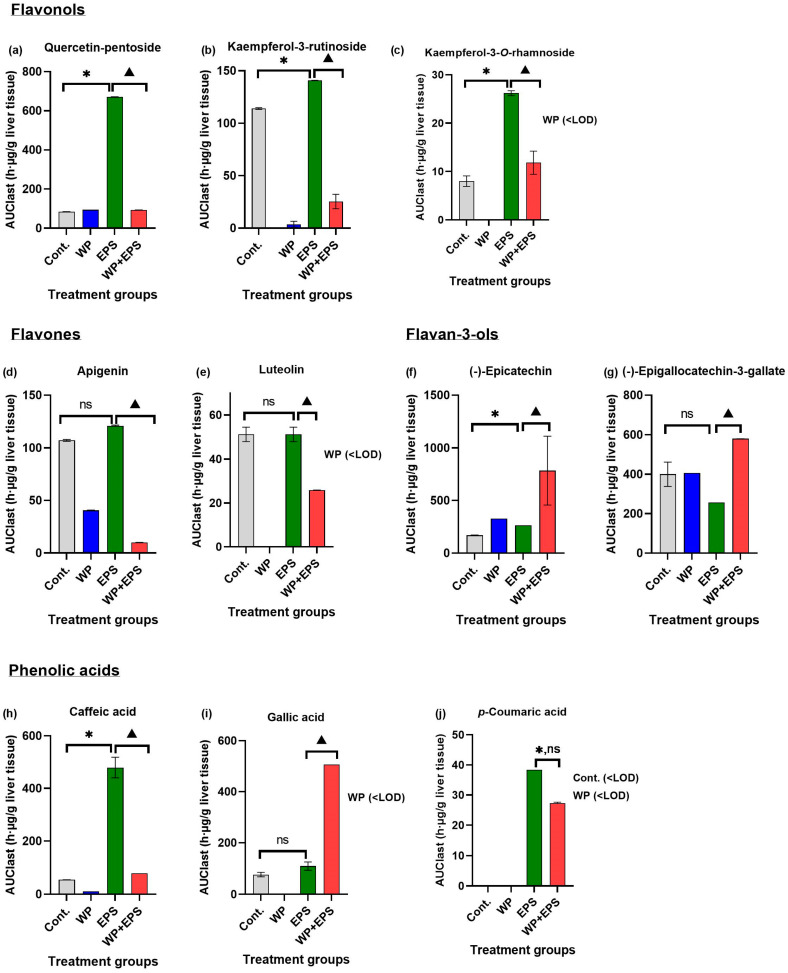
The liver area under the curve (AUC) values of polyphenols bioavailable after intragastric administration of blackthorn flower extract (25 mg/kg of body weight) and whey proteins (700 mg/kg of body weight) once daily for 28 days. The graphs depict polyphenolic compounds whose pharmacokinetic parameters (*c*_max_/AUC_last_) are statistically significantly different (*p* ≤ 0.05) between the EPS and EPS + WP groups. There were six animals per group (*n* = 6). * Statistically significant compared to the control group (*p* ≤ 0.05); ^▲^ statistically significant difference compared to the EPS group (*p* ≤ 0.05). The results are presented as the mean ± standard deviation. Abbreviations: Cont.—control; EPS—blackthorn flower water extract; WP—whey proteins; <LOD—below the limit of detection; ns—not statistically significant.

After subchronic dosing of blackthorn flower extract and whey proteins, a total of 26 polyphenolic compounds were detected in the liver, constituting 81.25% of the total number of polyphenolic compounds found in the aqueous extract of blackthorn flowers. A statistically significant difference in *c*_max_/AUC_last_ values (*p* ≤ 0.05) in the liver was observed for 10 polyphenolic compounds between the EPS + WP and EPS groups (31.25% of total N in EPS, 38.46% of liver detected) (Table 2, Figure 3 and Figure 4).

Analyzing the pharmacokinetic parameters of flavonols in the liver, the bioaccumulation of quercetin compounds in the EPS + WP group was generally lower compared to the EPS group. A statistically significantly lower concentration (*p* ≤ 0.05) of quercetin compounds was observed in the EPS + WP group for quercetin-pentoside (Figure 3a). The highest concentrations of quercetin compounds in the EPS + WP group were observed for quercetin-3-*O*-rutinoside (between days 1 and 7 of treatment), quercetin-rhamnoside, and quercetin-acetyl-hexoside (between days 1 and 14 of treatment). The biodynamics of other quercetin compounds in the EPS + WP group were similar to the EPS group but with different concentrations in the initial days of treatment. Among kaempferol compounds, the highest absorption values in the EPS + WP group were found for kampferol-pentoside (between days 7 and 14 of treatment), while statistically significantly lower concentrations (*p* ≤ 0.05) in the EPS + WP group compared to the EPS group were observed for kampferol-3-rutinoside and kampferol-3-*O*-rhamnoside (Figure 3b,c).

In contrast to flavonol compounds, the concentrations of detected flavan-3-ol compounds showed significantly higher absorbed concentration values. Statistically significantly higher (*p* ≤ 0.05) and relatively high concentrations in the EPS + WP group compared to the EPS group were achieved between days 1 and 14 of treatment for (−)-epigallocatechin-3-gallate and (−)-epicatechin-3-gallate compounds (Figure 3f,g).

The biodynamics of flavones apigenin and luteolin in the EPS + WP group were similar, and the values of absorbed concentrations remained in a very low concentration range throughout all 28 days of treatment (Figure 3d,e). Among phenolic acids, prominent concentrations in the EPS + WP group, although slightly lower compared to the EPS group, except for gallic acid, were observed for 4-*O*-p-coumaroylquinic acid, ferulic acid, and 4-*O*-caffeoylquinic acid (Figure 3i,j,h). The biodynamics of other acids were similar in the EPS and EPS + WP groups and remained in the low and very low concentration range.

### 3.3. Bioaccumulation of Polyphenols in the Kidneys after 28 Days of Treatment with Extract P. spinosa with a Whey-Protein-Enriched Diet

**Figure 5:**
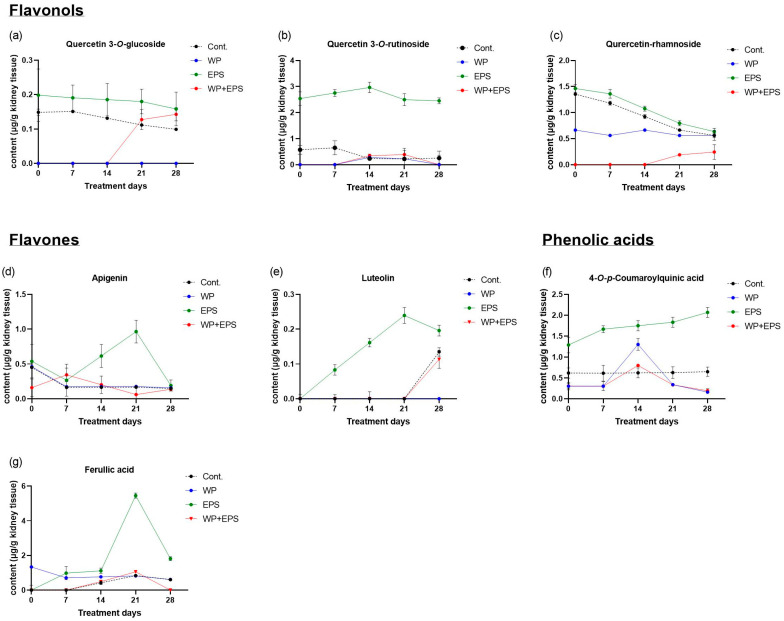
Pharmacokinetic curves of polyphenol bioavailability in the kidneys of C57BL/6 mice after intragastric administration of blackthorn flower extract (25 mg/kg of body weight) and whey protein (700 mg/kg of body weight) once daily for 28 days. The graphs show polyphenolic compounds with pharmacokinetic parameters (*c*_max_/AUC_last_) that are statistically significantly different (*p* ≤ 0.05) between the EPS and EPS + WP groups. Each group consisted of 6 animals. Results are presented as mean ± standard deviation. Abbreviations: Cont.—control; EPS—blackthorn flower water extract; WP—whey proteins; <LOD—below limit of detection.

**Figure 6:**
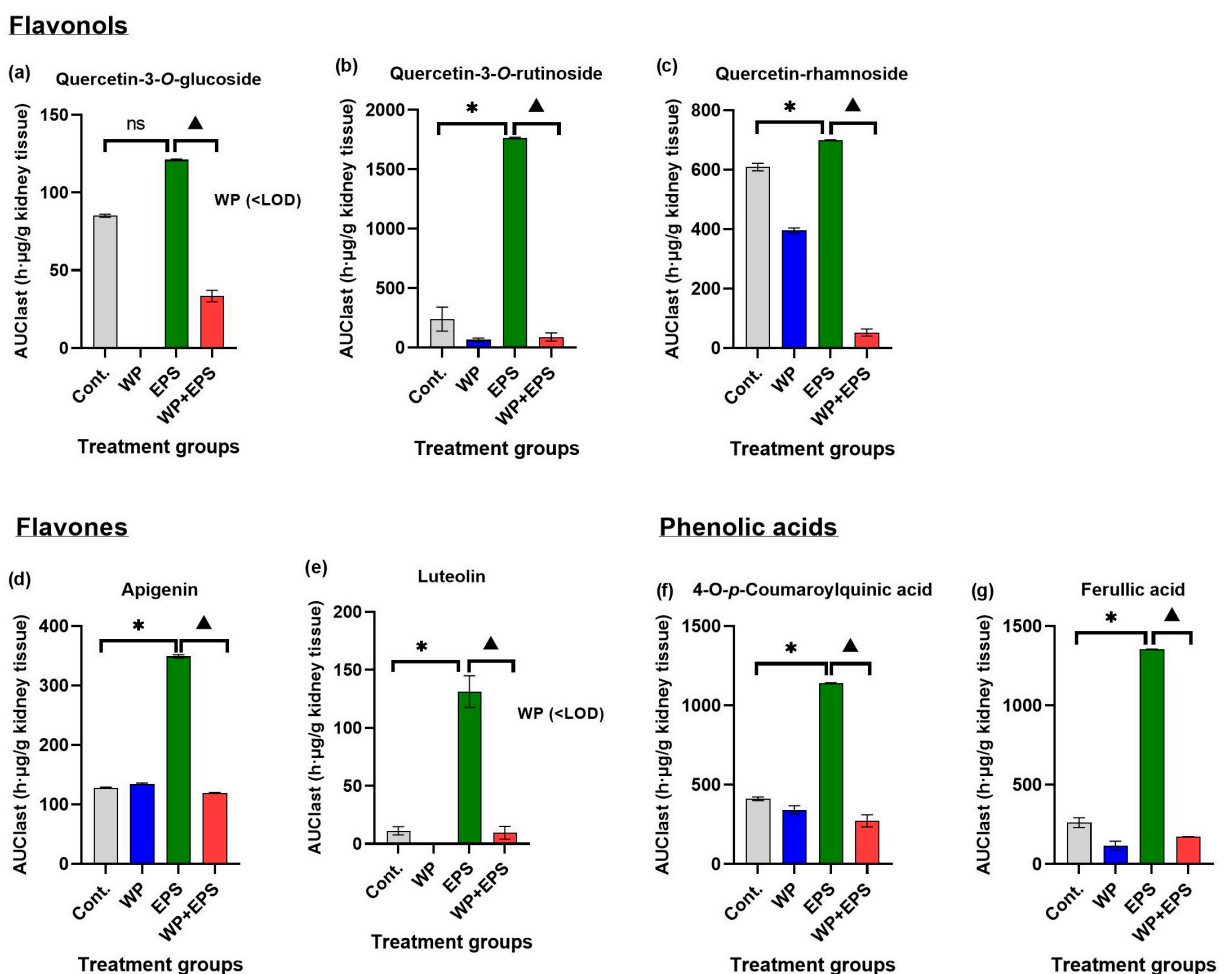
The kidney area under the curve (AUC) values for polyphenols’ bioavailability after intragastric administration of blackthorn flower extract (25 mg/kg of body weight) and whey protein (700 mg/kg of body weight) once daily for 28 days. The graphs depict polyphenolic compounds with pharmacokinetic parameters (*c*_max_/AUC_last_) that exhibit statistically significant differences (*p* ≤ 0.05) between the EPS and EPS + WP groups. Each group consisted of 6 animals. * Statistically significant compared to the control group (*p* ≤ 0.05); ^▲^ statistically significant compared to the EPS group (*p* ≤ 0.05). Results are presented as mean ± standard deviation. Abbreviations: Cont.—control; EPS—blackthorn flower water extract; WP—whey proteins; <LOD—below limit of detection.

After subchronic dosing of blackthorn flower extract and whey proteins, a total of 37.5% (N = 12/32) of polyphenolic compounds were detected in the kidneys compared to the total number of polyphenolic compounds found in the pure blackthorn flower extract. Statistically significant differences in *c*_max_/AUC_last_ were observed for seven compounds (21.8% of total N in EPS, 58.3% of kidneys detected) in the ECT + P group compared to the EPS group (Table 3, Figure 5 and Figure 6).

Regarding flavonol compounds, notable differences in bioavailability in the kidneys were observed for quercetin-3-*O*-rutinoside and quercetin-rhamnoside, with their concentrations being significantly lower in the EPS + WP group compared to the EPS group throughout the experimental animal treatment (Figure 5b,c). Similarly, kampferol-pentoside and quercetin-pentosyl-hexoside compounds showed similar temporal biodynamics between the EPS and EPS + WP groups, with higher concentrations observed for kampferol-pentoside.

For flavone compounds, statistically significant lower *c*_max/_AUC_last_ values (*p* ≤ 0.05) were found in the EPS + WP group compared to the EPS group. In contrast to the EPS group, the bioavailability of luteolin in the EPS + WP group was almost zero until day 21 of treatment, after which a slight increase in bioavailability was observed by day 28, though at very low concentrations (Figure 5e). As for apigenin, the highest concentrations, although within a very low concentration range, were observed between day 1 and day 7 of treatment in the EPS + WP group, after which its concentration decreased until day 21. Simultaneously, while the concentration of apigenin in the EPS + WP group decreased, it increased in the EPS group (Figure 5d).

For compounds (+)-catechin and (−)-EGCG, similar temporal biodynamics were observed between the EPS and EPS + WP groups. The most significant difference in bioabsorption between the EPS and EPS + WP groups was observed for (−)-epicatechin. The highest bioavailability in the EPS group was observed between day 1 and day 14 at moderate concentration levels throughout the treatment period. In contrast, in the EPS + WP group, (−)-epicatechin reached low concentrations between day 1 and day 14, after which its concentration increased to moderate levels by day 28.

When comparing the bioavailability of phenolic acids between the EPS and EPS + WP groups, the most significant differences in concentration were observed for ferulic acid, which reached high concentrations in the EPS group after day 14, unlike in the ECT + P group, where its concentration decreased after day 21 (Figure 5g). The biodynamics of 4-*O*-p-coumaroylquinic acid were similar between the EPS and EPS + WP groups until day 14, after which it decreased in the EPS + WP group and increased in the EPS group to moderate concentrations (Figure 5f).

### 3.4. Oxidative Stress Parameters in Small Intestine, Liver, and Kidney of Mice after 28 Days of Treatment with Extract P. spinosa with a Whey-Protein-Enriched Diet

The analysis of malondialdehyde (MDA) concentration in the small intestine, liver, and kidneys did not reveal any statistically significant differences (*p* ≤ 0.05) between the EPS and EPS + WP groups (Table 4).

A statistically significant increase (*p* ≤ 0.05) in catalase (CAT) enzyme activity was observed in the liver of the EPS + WP group compared to the EPS group and in the kidneys of the EPS group compared to the EPS + WP group (Table 4).

Analyzing the superoxide dismutase (SOD) enzyme activity, a statistically significant increase (*p* ≤ 0.05) in SOD activity was noticed in the small intestine of the WP group compared to the WP + EPS group and in the liver of the EPS + WP group compared to the EPS group. Additionally, in the kidneys, a statistically significant decrease (*p* ≤ 0.05) in SOD concentration was observed in the EPS + WP group compared to the EPS group (Table 4).

Regarding the concentration of total glutathione (tGSH), statistically significantly higher (*p* ≤ 0.05) tGSH levels were observed in the liver of the ECT + WP group compared to the ECT group. Conversely, in the small intestine, there was a statistically significant lower concentration of tGSH in the ECT + WP group compared to the ECT group (Table 4).

## 4. Discussion

Research on the bioavailability of polyphenols is not straightforward, as numerous factors influence their absorption and bioavailability [38]. Some of these factors include the interactions with the plant matrix, physical and chemical characteristics of polyphenols, luminal pH, metabolic processes involving intestinal and hepatic enzymes, and gut microbiota [39]. Furthermore, polyphenol bioavailability can be affected by interactions with food matrices, as well as the bolus of food and fluids released by the gastrointestinal tract during the digestion process [40]. In vitro studies indicate that nutrients surrounding polyphenols within the digestive tract have a significant impact on their bioavailability and, consequently, their biological activity [41]. In this study, we investigated the influence of whey protein, the most commonly used source of protein in a population of exercisers, on the bioavailability of 32 polyphenolic compounds from the aqueous extract of blackthorn flower in an in vivo mouse model (C57BL/6). The whey protein has an advantage over other proteins due to its rapid absorption in the intestines, digestibility, and practical intake, ultimately resulting in protein synthesis for muscle building, maintenance of existing body mass, and regeneration [42].

Numerous physiological and technological in vitro studies describe the interactions between polyphenols and proteins, and these interactions can have both positive and negative effects on polyphenol bioavailability [43]. The most common interactions that play a dominant role in the formation of protein–polyphenol complexes are hydrogen bonds, hydrophobic interactions, and van der Waals forces [44,45]. Phenolic groups in polyphenols are good hydrogen bond donors and can form hydrogen bonds with the C=O groups in proteins. Hydrogen bonds can also be formed between the hydroxyl (-OH) groups of phenolic compounds and the hydroxyl (-OH) or amino (-NH2) groups of proteins [46,47]. Hydrophobic interactions mainly occur between the nonpolar aromatic rings of polyphenols and the hydroxyl groups of amino acids in proteins, while van der Waals forces act between all types of molecules and can contribute to the overall formation of protein–polyphenol complexes [48,49]. In a recent study by Chima et al. [50], the physicochemical interactions between blueberry polyphenols and proteins from dairy and plant sources were investigated in vitro, along with the impact of polyphenol complexation on protein digestion. They compared whey proteins with pea and hemp proteins. The results of their study indicate that all three protein sources form complexes with blueberry polyphenols, with pea and hemp proteins showing a higher affinity for binding polyphenols than whey proteins. This difference in binding affinity could be attributed to observed variations in the secondary structure of the proteins. The authors also concluded that the addition of polyphenols did not affect the in vitro digestion of any of the studied proteins and that pH values might play a role in the formation of complexes between polyphenols and proteins.

In this study, in the small intestine, a total of 25 polyphenolic compounds were detected in the group treated with blackthorn flower extract in combination with whey proteins (EPS + WP), which is two compounds less compared to the group treated with pure aqueous blackthorn flower extract (EPS). A statistically significant decrease (*p* ≤ 0.05) in AUC_last_/*c*_max_ in the small intestine was observed for 11 polyphenolic compounds in the EPS + WP group compared to the EPS group (Table 1, Figure 1 and Figure 2). Liver analysis did not reveal a difference in the number of detected compounds between the EPS + WP and EPS groups. Out of a total of 26 detected polyphenolic compounds, a statistically significant decrease in AUC_last_/*c*_max_ was found for 8 compounds in the EPS + WP group, while for 2 compounds, a statistically significant increase in AUC or *c*_max_ was observed compared to the EPS group (*p* ≤ 0.05) (Table 2, Figure 3 and Figure 4). Kidney analysis did not show differences in the number of detected polyphenolic compounds. Out of a total of 12 detected compounds in the kidney, a statistically significant decrease in AUC or *c*_max_ was observed for 7 polyphenolic compounds in the EPS + WP group compared to the EPS group (*p* ≤ 0.05) (Table 3, Figure 5 and Figure 6).

Among all polyphenolic compounds, the highest bioabsorption in the group treated with aqueous blackthorn flower extract in combination with whey proteins (EPS + WP) was observed in the liver for compounds from the flavan-3-ol group: (−)-epicatechin (EC) and (−)-epigallocatechin-3-gallate (EGCG). The *c*_max_ and AUC_last_ values for EC were three times higher, and *t*_max_ was half the value in the EPS + WP group (*c*_max_ EPS + WP = 4.56 µg/g; *t*_max_ = 168 h; AUC_last_ = 783.4 h·µg/g) compared to the EPS group (*c*_max_ EPS = 1.40 µg/g; *t*_max_ = 336 h; AUC_last_ = 265.9 h·µg/g). Similar pharmacokinetic parameter values were observed for the EGCG compound, where the *c*_max_ and AUCl_ast_ values were almost 2.5 times higher, and *t*_max_ was half the value in the EPS + WP group (*c*_max_ EPS + WP = 2.18 µg/g; *t*_max_ = 168 h; AUC_last_ = 596.3 h·µg/g) compared to the EPS group (*c*_max_ EPS = 0.88 µg/g; *t*_max_ = 336 h; AUC_last_ = 256.6 h·µg/g) (Table 2, Figure 3 and Figure 4), indicating a higher absorption and bioavailability of EC and EGCG when consumed in combination with whey proteins. Previous research on human CaCo-2 cells did not identify specific receptors that transport EC and EGCG into cells, so it is believed that the mechanism of catechin molecule transfer is mainly based on passive diffusion, including paracellular and transcellular diffusion. In vitro studies on cell cultures also suggest that after absorption, EGCG undergoes active efflux through ATP-dependent proteins. EGCG and its metabolites serve as common substrates for the ABC protein family, including multidrug resistance proteins (MRP) and P-glycoprotein (P-gp), through which most absorbed catechins are pumped back into the extracellular or intestinal space, limiting their bioavailability [51]. Xiue et al. [52] studied the interactions between EGCG and whey protein isolates and the effect of their interactions on the conformational and functional changes of whey proteins by using FTIR spectra. Their research confirms that EGCG forms a higher proportion of covalent bonds with whey proteins compared to non-covalent bonds, which modify the secondary structure of whey proteins, improving their functional properties. Furthermore, Xuejiao et al. [53] investigated the effect of competitive interactions between tea catechins, milk proteins, and digestive enzymes on protein digestibility, catechin bioavailability, and antioxidant activity by simulating digestion in vitro. The inhibitory effect of catechins on digestive enzymes was positively correlated with the binding affinity of catechins to digestive enzymes. The interaction between tea catechins and milk proteins or digestive enzymes resulted in reduced protein digestibility. The bioavailability of catechins and the antioxidant activity of tea with milk were reduced by the interaction between proteins and catechins but increased by the competition between proteins, catechins, and digestive enzymes. After the addition of β-lactoglobulin (β-Lg), the bioavailability of EGCG increased by 252.6%, epigallocatechin (EGC) by 37.0%, and EC by 37%. The results of their research indicate that increased bioavailability and antioxidant activity are positively correlated with the binding affinity of catechins and proteins. Furthermore, previous in vitro studies suggest that milk proteins (casein, whey proteins), as well as other types of proteins such as zein proteins from corn, soy, and rice bran protein isolates, enhance the stability, permeability, and bioavailability of catechins and can serve as ideal carriers for delivering catechin group molecules [54,55,56,57]. The results of this in vivo study can be compared to the aforementioned in vitro studies on increased absorption and bioavailability of flavan-3-ols when consumed with whey proteins.

In a study conducted by Świeca et al. [58], it was observed that protein-rich bread reduces the bioavailability and total antioxidant capacity of polyphenols extracted from onion skin with a high content of flavonoids, especially quercetin. The reduced bioavailability is attributed to the formation of complexes between polyphenols and proteins through hydrophobic interactions and hydrogen bonds. A similar study was conducted by Oksuz et al. [59], who investigated the effect of dairy products (skim milk, non-fat yogurt, probiotic yogurt) on the bioavailability of cherry polyphenols using a simulated gastrointestinal digestion method. The results of their study also describe a reduction in the bioavailability of certain polyphenols, especially rutin and cyanidin-3-*O*-glucoside, which they attribute to interactions and high binding affinity between polyphenols and dairy proteins. Some studies have examined changes in the bioavailability of anthocyanins due to their interaction with proteins in different food matrices. Mullen et al. [60] studied the bioavailability of pelargonidin-3-*O*-glucoside from strawberries consumed with or without cream in vivo in plasma and urine samples. They concluded that there were no statistically significant differences in the digestion of strawberries with cream and strawberries alone. In contrast, Serafini et al. [61] investigated the bioavailability and antioxidant capacity of polyphenols in plasma after consuming blueberries (*Vaccinium corymbosum* L.) with and without milk. They conducted a crossover study with 11 healthy volunteers who consumed 200 g of blueberries with 200 mL of water or 200 mL of milk. Plasma samples were collected 1, 2, and 5 h after consumption. The results of this study revealed that blueberry intake increased plasma antioxidant capacity (+6.1%, *p* < 0.001; +11.1%, *p* < 0.05). However, when milk and blueberries were consumed together, plasma antioxidant capacity did not increase. The consumption of blueberries in combination with milk attenuated the in vivo antioxidant activity of blueberries. Similar research results were obtained in an in vitro study by Sengul et al. [62], where the impact of food components (meat proteins, soy proteins, gluten, casein) on the bioavailability and antioxidant capacity of pomegranate polyphenols was also investigated by simulating gastrointestinal digestion. After post-gastric digestion, the proteins that were used reduced the total antioxidant capacity of polyphenols. Furthermore, Rodríguez-Roque et al. [63] studied the bioavailability of polyphenols in fruit juice and a combination of fruit juice with milk using an in vitro model. The results of their research indicate that adding milk increased the bioavailability of lipophilic compounds but not hydrophilic compounds.

Budryn et al. [64] investigated the interactions of *p*-coumaric, ferulic, caffeic, and chlorogenic acids from green coffee with egg white, whey, and soy proteins, depending on pH and temperature. The results of their research suggest a weak release of phenolic acids from the phenolic acid–protein complex after simulating in vitro digestion, which results in reduced availability of their free forms for absorption. Alaa Hamed Ibrahim [65] studied the interactions between polyphenols (*p*-coumaric acid, gallic acid, caffeic acid, and chlorogenic acid) with camel’s milk casein and whey proteins. The research results confirmed that phenolic acids exhibit a stronger binding affinity with casein than with whey proteins. Additionally, the research results indicated that with an increase in the molecular weight of polyphenols, the binding percentage to proteins increased in the following order: *p*-coumaric acid < gallic acid < caffeic acid < chlorogenic acid. After two phases of in vitro digestion, it was observed that all phenolic acids had a lower binding affinity to camel casein than to whey proteins, indicating that casein complexes from camel’s milk are more easily digested compared to the phenolic acid and whey protein complexes during in vitro simulated digestion.

In this study, among the flavonol polyphenolic compounds, a statistically significant decrease in AUC or *c*_max_ (*p* ≤ 0.05) was observed in the EPS + WP group compared to the EPS group in the small intestine for five flavonol compounds (quercetin-pentoside, quercetin-rhamnoside, kaempferol-3-rutinoside, kaempferol-pentoside, kaempferol-3-rhamnoside), in the liver for three compounds (quercetin-pentoside, kaempferol-3-rutinoside, and kaempferol-3-*O*-rhamnoside), and in the kidneys for three compounds (quercetin-3-*O*-glucoside, quercetin-3-*O*-rutinoside, and quercetin-rhamnoside). Among the flavone compounds, statistically significantly lower AUC/*c*_max_ values (*p* ≤ 0.05) in the EPS + WP group compared to the EPS group were found for the compounds apigenin and luteolin in all three organs. Furthermore, in the analysis of polyphenolic compounds from the group of phenolic acids, statistically significant lower AUC or *c*_max_ (*p* ≤ 0.05) in the EPS + WP group compared to the EPS group were observed in the small intestine for 3-*O*-feruloylquinic, 4-*O*-*p*-coumaroylquinic, and gallic acid; in the liver for caffeic, gallic, and *p*-coumaric acid; and in the kidneys for 4-*O*-*p*-coumaroylquinic and ferulic acid. From these results, it can be concluded that a subchronic intake of polyphenols in combination with whey protein leads to statistically significant changes in the bioavailability of certain compounds from the flavonol, flavone, and phenolic acid groups detected in the aqueous extract of hawthorn flower. Although there were no statistically significant changes in pharmacokinetic parameters (AUC/*c*_max_) for other types of polyphenols from the flavonol and phenolic acid groups, the results of this study clearly show that the majority of detected polyphenols from the flavonol and phenolic acid groups in the EPS + WP group exhibit a longer time period (*t*_max_) to reach their maximum concentrations, as well as a smaller AUC_last_, indicating their reduced exposure in the organ. The results of this study also demonstrate that whey proteins and certain compounds from the flavonol, flavone, and phenolic acid groups engage in interactions that can affect the kinetics of absorption of certain polyphenolic compounds and, consequently, their bioavailability. These results can be compared to the studies described earlier and other works that used different sources of polyphenols.

So far, published research on polyphenols from the blackthorn plant and whey proteins provides evidence of their positive antioxidant effects on various organic systems in both animal and human organisms. In an in vivo study by Balta et al. [9], it was proven that polyphenols from blackthorn flower extract and their metabolites, in the group treated with blackthorn flower extract, compared to the control group, reduce the concentration of malondialdehyde (MDA) and activate enzymes (GSH, CAT, SOD) involved in the antioxidant defense system. Additionally, in a study by Pozzo et al. [66], it was demonstrated that blackthorn fruit extract reduces hemolysis of human erythrocytes and contributes to reduced oxidative stress in the liver and brain of rats induced by a high-fat diet and hyperglycemia. Furthermore, Wael. I. et al. [67] investigated the antioxidant and hypolipidemic effect of whey protein on radiation-induced damage in rats. The rats were divided into five groups: a control group, groups exposed to radiation of 5 and 10 Gy (gray), and groups orally administered whey protein after exposure to radiation of 5 and 10 Gy. Gamma radiation caused decreased levels of hematological parameters, tGSH, SOD, CAT, total antioxidant capacity, and increased MDA concentration. The authors also reported that the group of rats that received whey proteins after radiation had an improved antioxidant status, and whey proteins minimized changes in lipids and hematological parameters induced by gamma radiation. Veskoukis et al. [68] investigated the antioxidant effect of sheep whey protein in different rat organs. Rats received a daily water solution of protein at a dose of 1g/kg of body weight/day for 28 days. Whey proteins improved the antioxidant profile of the liver, small intestine, lungs, and muscles but did not affect the kidney status. The results of their study were based on changes observed in the expression of glutamate-cysteine ligase, catalase, and SOD-1 proteins measured in all tissues and glutathione S-transferase activity assessed in muscles. Although the action of whey proteins is tissue-specific, the authors concluded that the effect of whey protein is biologically beneficial and can serve as a biofunctional food ingredient that can improve the redox profile when applied against diseases related to oxidative stress. The results of this research also demonstrated that the polyphenols from the aqueous extract of blackthorn flower, when consumed in combination with whey proteins, compared to the group receiving pure blackthorn flower extract, lead to the increase in the concentration of antioxidant enzymes (tGSH, SOD, CAT) in the mouse liver. The elevated levels of antioxidant enzymes in the ESP + WP group can be attributed to whey proteins, which provide a rich source of amino acids for the synthesis of antioxidant enzymes, as well as to the polyphenols quercetin-3-*O*-rutinoside, kampferol-pentoside, (−)-epicatechin-3-gallate, EGCG, and 4-*O*-caffeoylquinic acid, which were found to have the highest bioaccumulated concentrations in the liver tissue. Summarizing the overall results obtained from blackthorn flower extract in combination with whey proteins, it can be concluded that this combination has a biologically beneficial effect of increasing the absorption and bioaccumulation of certain polyphenolic molecules. It can serve as a biofunctional food ingredient that can improve the overall organism’s redox profile and affect metabolic pathways, leading to health benefits and the prevention of diseases related to oxidative stress.

## 5. Conclusions

In conclusion, the results of this in vivo study indicate increased bioabsorption and bioavailability of flavan-3-ols (EC, EGCG) and reduced absorption kinetics of certain polyphenolic compounds from the group of flavonols, flavones, and phenolic acids in the organs of C57BL/6 mice after intragastric administration of blackthorn flower water extract in combination with a diet enriched with whey proteins. Additionally, subchronic intake of polyphenols from the blackthorn flower aqueous extract in combination with a diet enriched with whey proteins induces the synthesis of total glutathione (tGSH) in the liver and superoxide dismutase (SOD) in the liver and small intestine. The results of this study suggest potential applications in the development of functional foods aimed at achieving optimal health of an organism and reducing the risk of disease development.

## Figures and Tables

**Table 1:** The UPLC analysis and fundamental pharmacokinetic parameters of the detected polyphenols in the small intestine in the group treated with blackthorn flower extract in combination with a protein-enriched diet daily for 28 days.

^a^ Small Intestine Bioavailability		1–28 Day, Subchronic Dose *		
Compound	Group	*c*_max_ (µg/g Tissue)	*t*_max_ (h)	AUC_last_ (h·µg/g Tissue)
Flavonols				
Isorhamnetin-3-rutinoside	Cont.	0.39 ± 0.01	24 ± 2	252.9 ± 1.5
	WP	0.41 ± 0.00	504 ± 0	263.9 ± 0.0
	EPS	0.43 ± 0.25	24 ± 0	273.2 ± 4.5
	WP + EPS	0.41 ± 0.00	24 ± 0	263.1 ± 0.0
Quercetin-3-*O*-glucoside	Cont.	0.17 ± 0.00	168 ± 0	63.9 ± 10.2
	WP	0.55 ± 0.00	168 ± 0	115.1 ± 1.1
	EPS	0.56 ± 0.05 *	504 ± 19	151.0 ± 35.5 *
	WP + EPS	0.38 ± 0.00	336 ± 0	88.9 ± 0.5
Quercetin-3-*O*-rutinoside	Cont.	0.78 ± 0.00	168 ± 0	270.3 ± 4.3
	WP	1.36 ± 0.00	24 ± 8	546.2 ± 1.0
	EPS	1.73 ± 0.21 *	24 ± 3	952.0 ± 120.8 *
	WP + EPS	1.40 ± 0.03	24 ± 2	909.1 ± 114.6
Quercetin-acetyl-hexoside	Cont.	0.52 ± 0.01	672 ± 291	219.2 ± 23.8
	WP	0.54 ± 0.00	504 ± 0	305 ± 0.0
	EPS	0.67 ± 0.39	672 ± 0	429.9 ± 0.9 *
	WP + EPS	0.56 ± 0.00	168 ± 0	352.4 ± 0.0
Quercetin-pentoside	Cont.	1.37 ± 0.07	168 ± 83	654.5 ± 19.2
	WP	0.14 ± 0.00	504 ± 0	93.5 ± 1.1
	EPS	1.94 ± 1.03	24 ± 0	814.6 ± 8.0
	WP + EPS	0.14 ± 0.00 ^▲^	336 ± 0	93.6 ± 5.2 ^▲^
Quercetin-pentosyl-hexoside	Cont.	0.34 ± 0.01	24 ± 8	152.3 ± 13.9
	WP	0.30 ± 0.00	672 ± 0	126.7 ± 0.5
	EPS	0.45 ± 0.04	24 ± 0	179.9 ± 10.2
	WP + EPS	0.35 ± 0.04	336 ± 0	153.5 ± 25.4
Qurercetin-rhamnoside	Cont.	0.96 ± 0.10	336 ± 83	387.7 ± 11.8
	WP	1.68 ± 0.00	672 ± 0	235.2 ± 1.2
	EPS	1.68 ± 0.95 *	24 ± 0	723.7 ± 17.3 *
	WP + EPS	1.52 ± 0.70	672 ± 97	213.2 ± 131.2 ^▲^
Kaempferol-3-glucoside	Cont.	0.16 ± 0.00	168 ± 83	93.5 ± 2.1
	WP	0.22 ± 0.00	672 ± 0	89.0 ± 0.0
	EPS	0.30 ± 0.17 *	168 ± 0	146.1 ± 3.8 *
	WP + EPS	0.16 ± 0.00	336 ± 0	87.6 ± 0.0
Kaempferol-3-rutinoside	Cont.	0.24 ± 0.00	168 ± 0	116.3 ± 3.2
	WP	<LOD	-	-
	EPS	0.28 ± 0.16	168 ± 83	132.1 ± 1.2
	WP + EPS	<LOD ^▲^	-	-
Kaempferol-pentoside	Cont.	2.75 ± 0.00	168 ± 0	1344.3 ± 6.0
	WP	2.55 ± 0.00	672 ± 0	1282.6 ± 0.0
	EPS	5.29 ± 2.91 *	24 ± 0	1559.4 ± 74.7
	WP + EPS	2.10 ± 0.00 ^▲^	168 ± 0	1071.4 ± 0.0
Kaempferol-3-*O*-rhamnoside	Cont.	0.00 ± 0.00	168 ± 0	0.0 ± 0.0
	WP	<LOD	-	-
	EPS	0.06 ± 0.00	672 ± 0	9.9 ± 0.0 *
	WP + EPS	0.00 ± 0.00 ^▲^	24 ± 0	0.0 ± 0.0 ^▲^
Kaempferol-rhamnosyl-hexoside	Cont.	0.69 ± 0.02	24 ± 8	334.8 ± 4.9
	WP	0.68 ± 0.00	672 ± 0	300.1 ± 0.0
	EPS	0.84 ± 0.49	24 ± 0	384.6 ± 3.5
	WP + EPS	0.54 ± 0.00	336 ± 0	286.7 ± 0.0
Kaempferol-acetyl- hexoside	Cont.	0.04 ± 0.01	504 ± 0	13.3 ± 4.7
	WP	0.04 ± 0.00	24 ± 0	16.3 ± 0.0
	EPS	0.05 ± 0.05	672 ± 37	7.6 ± 2.7
	WP + EPS	0.06 ± 0.00	672 ± 0	24.7 ± 0.0
Kaempferol-pentosyl-hexoside	Cont.	0.51 ± 0.01	168 ± 83	260.6 ± 13.9
	WP	0.51 ± 0.00	672 ± 0	242.4 ± 0.0
	EPS	0.85 ± 0.45 *	24 ± 0	329.4 ± 10.2
	WP + EPS	0.55 ± 0.00	336 ± 0	266.2 ± 0.0
Flavones				
Apigenin	Cont.	0.89 ± 0.14	24 ± 0	188.3 ± 17.8
	WP	0.08 ± 0.01	24 ± 14	1.1 ± 0.74
	EPS	2.50 ± 0.25 *	24 ± 3	523.9 ± 72.5 *
	WP + EPS	<LOD ^▲^	-	-
Luteolin	Cont.	0.20 ± 0.10	168 ± 83	89.5 ± 19.2
	WP	0.20 ± 0.00	504 ± 0	17.5 ± 0.2
	EPS	0.64 ± 0.28 *	24 ± 3	171.8 ± 30.6 *
	WP + EPS	0.22 ± 0.02 ^▲^	504 ± 388	18.8 ± 2.0 ^▲^
Flavan-3-ols				
(+)-Catechin	Cont.	0.13 ± 0.00	672 ± 374	83.5 ± 0.5
	WP	0.23 ± 0.00	168 ± 0	102.3 ± 0.2
	EPS	0.16 ± 0.09	672 ± 0	86.8 ± 1.5
	WP + EPS	0.13 ± 0.00	168 ± 83	87.2 ± 11.2
(−)-Epicatechin	Cont.	0.05 ± 0.04	168 ± 97	9.4 ± 1.1
	WP	0.08 ± 0.00	504 ± 0	7.5 ± 0.2
	EPS	0.06 ± 0.03	168 ± 0	12.5 ± 0.0
	WP + EPS	0.09 ± 0.03	504 ± 83	8.3 ± 1.5
(−)-Epigallocatechin-3-gallate (EGCG)	Cont.	0.02 ± 0.00	504 ± 291	6.3 ± 0.8
	WP	0.05 ± 0.00	672 ± 0	9.6 ± 0.1
	EPS	0.17 ± 0.02 *	672 ± 0	14.5 ± 13.9 *
	WP + EPS	0.05 ± 0.01 ^▲^	168 ± 0	3.9 ± 1.3 ^▲^
Phenolic acids				
3-*O*-Feruloylquinic acid	Cont.	0.62 ± 0.13	168 ± 83	211.9 ± 37.3
	WP	0.44 ± 0.00	672 ± 0	155.6 ± 0.0
	EPS	4.61 ± 0.42 *	168 ± 37	768.4 ± 6.3 *
	WP + EPS	1.40 ± 0.00 ^▲^	336 ± 0	471.7 ± 0 ^▲^
3-*O*-*p*-Coumaroylquinic acid	Cont.	0.00 ± 0.05	24 ± 8	0.0 ± 6.2
	WP	0.05 ± 0.00	24 ± 0	22.4 ± 0.0
	EPS	0.20 ± 0.02	168 ± 14	71.8 ± 14.4 *
	WP + EPS	0.28 ± 0.00	336 ± 0	81.5 ± 0.0
4-*O*-Caffeoylquinic acid	Cont.	1.64 ± 0.10	672 ± 374	896.5 ± 83.2
	WP	1.54 ± 0.00	504 ± 0	953.5 ± 0.0
	EPS	1.81 ± 1.17	168 ± 34	1110.1 ± 25.4
	WP + EPS	1.50 ± 0.00	24 ± 0	910.6 ± 0.0
4-*O*-*p*-Coumaroylquinic acid	Cont.	1.22 ± 0.05	24 ± 0	627.7 ± 4.2
	WP	0.95 ± 0.00	168 ± 0	623.6 ± 4.0
	EPS	2.16 ± 1.27 *	504 ± 97.	1095.0 ± 27.8 *
	WP + EPS	0.93 ± 0.00 ^▲^	168 ± 0	570 ± 160.7 ^▲^
Caffeic acid	Cont.	0.20 ± 0.05	168 ± 0	41.6 ± 17.4
	WP	0.20 ± 0.00	672 ± 0	62.7 ± 0.0
	EPS	0.37 ± 0.21 *	168 ± 0	111.8 ± 13.9 *
	WP + EPS	0.22 ± 0.00	504 ± 0	108.2 ± 0.0
Ferullic acid	Cont.	1.04 ± 0.30	504 ± 0	368.3 ± 61.5
	WP	1.57 ± 0.00	504 ± 0	823.5 ± 1.2
	EPS	1.68 ± 0.95 *	24 ± 2	740.9 ± 9.7 *
	WP + EPS	1.54 ± 0.22	672 ± 374	847.4 ± 113.8
Gallic acid	Cont.	1.28 ± 0.67	24 ± 0	215.1 ± 96.7
	WP	0.63 ± 0.00	672 ± 0	183.6 ± 0.0
	EPS	2.21 ± 1.27 *	168 ± 0	575.2 ± 12.6 *
	WP + EPS	0.42 ± 0.00 ^▲^	336 ± 0	191.6 ± 0.0 ^▲^
*p*-Coumaric acid	Cont.	0.48 ± 0.14	504 ± 277	186.7 ± 37.4
	WP	0.57 ± 0.00	24 ± 0	302 ± 0.0
	EPS	1.67 ± 0.94 *	24 ± 0	444.9 ± 6.3 *
	WP + EPS	1.22 ± 0.00	504 ± 0	567.6 ± 0.0

^a^ The mice were treated intragastrically with blackthorn flower water extract (EPS) at a dose of 25 mg/kg of body weight per day and whey proteins (WP) at a dose of 700 mg/kg of body weight per day. There were six animals per group (*n* = 6). * Statistically significant difference compared to the control group (*p* ≤ 0.05); ^▲^ statistically significant difference compared to the EPS group (*p* ≤ 0.05). The results are presented as the mean ± standard deviation. Abbreviations: Cont.—control; EPS—blackthorn flower water extract; WP—whey proteins; *c*_max_—maximal concentration of the compound in tissue; *t*_max_—average time when the compound reaches *c*_max_; AUC_last_—area under the curve; <LOD—below the limit of detection.

**Table 2:** UPLC analysis and basic pharmacokinetic parameters of detected polyphenols in the liver in the groups treated with blackthorn flower extract in combination with a protein-enriched diet daily for 28 days.

^a^ Liver Bioavailability		1–28 Day, Subchronic Dose *		
Compound	Group	*c*_max_ (µg/g Tissue)	*t*_max_ (h)	AUC_last_ (h·µg/g Tissue)
Flavonols				
Isorhamnetin-3-rutinoside	Cont.	0.39 ± 0.00	168 ± 29	248.0 ± 1.2
	WP	0.43 ± 0.00	672 ± 356	268.7 ± 0.0
	EPS	0.41 ± 0.00	168 ± 83	265.4 ± 0.2
	WP + EPS	0.40 ± 0.02	504 ± 0	266.9 ± 0.0
Quercetin-3-*O*-glucoside	Cont.	0.14 ± 0.09	24 ± 0	70.6 ± 0.8
	WP	0.32 ± 0.35	336 ± 0	107.2 ± 0.0
	EPS	0.14 ± 0.09	24 ± 0	70.6 ± 0.8
	WP + EPS	0.65 ± 0.02	168 ± 0	171.4 ± 0.0
Quercetin-3-*O*-rutinoside	Cont.	1.55 ± 0.32	336 ± 83	715.1 ± 78.9
	WP	1.51 ± 0.04	168 ± 0	777.9 ± 6.6
	EPS	2.57 ± 0.03 *	336 ± 83	1113.1 ± 0.6 *
	WP + EPS	1.79 ± 0.04	168 ± 0	845.1 ± 24.8
Quercetin-acetyl-hexoside	Cont.	0.55 ± 0.00	24 ± 3	302.8 ± 2.5
	WP	0.55 ± 0.00	168 ± 0	355.7 ± 0.0
	EPS	0.64 ± 0.00	24 ± 3	419.2 ± 0.2
	WP + EPS	1.17 ± 0.04	24 ± 0	500.5 ± 0.0
Quercetin-pentoside	Cont.	0.13 ± 0.00	168 ± 0	84.2 ± 1.1
	WP	0.14 ± 0.00	168 ± 0	94.5 ± 0.0
	EPS	1.44 ± 0.02 *	168 ± 83	670.0 ± 1.6 *
	WP + EPS	0.14 ± 0.11 ^▲^	336 ± 37.2	92.7 ± 0.7 ^▲^
Quercetin pentosyl-hexoside	Cont.	0.36 ± 0.02	168 ± 0	157.7 ± 0.8
	WP	0.12 ± 0.00	168 ± 0	43.6 ± 0.0
	EPS	0.47 ± 0.06	168 ± 0	207.5 ± 0.2
	WP + EPS	0.31 ± 0.02	504 ± 0	141.5 ± 0.0
Qurercetin-rhamnoside	Cont.	1.95 ± 0.03	168 ± 0	606.7 ± 1.6
	WP	1.68 ± 0.1	168 ± 0	259.3 ± 0.0
	EPS	1.67 ± 0.01	168 ± 0	635.4 ± 1.0
	WP + EPS	1.52 ± 0.17	336 ± 0	659.3 ± 0.2
Kaempferol-3-glucoside	Cont.	0.16 ± 0.00	24 ± 0	71.6 ± 0.4
	WP	0.06 ± 0.00	24 ± 0	17.6 ± 0.0
	EPS	0.26 ± 0.01	168 ± 0	108.3 ± 0.3
	WP + EPS	0.15 ± 0.1	336 ± 0	71.6 ± 0.2
Kaempferol-3-rutinoside	Cont.	0.23 ± 0.01	24 ± 8	114.9 ± 0.8
	WP	0.04 ± 0.00	672 ± 0	3.7 ± 2.9
	EPS	0.29 ± 0.00 *	168 ± 0	140.8 ± 0.1 *
	WP + EPS	0.06 ± 0.01 ^▲^	336 ± 29	25.4 ± 6.9 ^▲^
Kampferol-pentoside	Cont.	2.60 ± 0.00	168 ± 0	1243. 4 ± 1.1
	WP	2.58 ± 0.00	168 ± 0	1230.1 ± 0.0
	EPS	2.69 ± 0.00	168 ± 0	1382.3 ± 1.0
	WP + EPS	2.67 ± 0.14	336 ± 0	1416.4 ± 0.0
Kaempferol-3-*O*-rhamnoside	Cont.	0.03 ± 0.00	168 ± 34	8.0 ± 1.1
	WP	<LOD	-	-
	EPS	0.12 ± 0.02 *	168 ± 0	26.2 ± 0.5 *
	WP + EPS	0.03 ± 0.00 ^▲^	336 ± 0	11.8 ± 2.4 ^▲^
Kaempferol-rhamnosyl- hexoside	Cont.	0.70 ± 0.02	168 ± 83	330.5 ± 1.5
	WP	0.26 ± 0.01	168 ± 0	134.9 ± 0.5
	EPS	0.80 ± 0.00	168 ± 0	374.2 ± 0.59
	WP + EPS	0.62 ± 0.04	336 ± 290	392.8 ± 0.8
Kaempferol-acetyl-hexoside	Cont.	0.02 ± 0.00	168 ± 96	4.1 ± 0.8
	WP	0.13 ± 0.00	504 ± 0	44.8 ± 0.0
	EPS	0.07 ± 0.00 *	168 ± 0	13.4 ± 0.0 *
	WP + EPS	0.05 ± 0.00	24 ± 0	22 ± 0.2
Flavones				
Apigenin	Cont.	0.28 ± 0.01	24 ± 8	107.2 ± 0.9
	WP	0.50 ± 0.00	168 ± 0	40.6
	EPS	0.20 ± 0.03	168 ± 27	121.1 ± 0.5
	WP + EPS	0.10 ± 0.03 ^▲^	24 ± 0	9.9 ± 0.1 ^▲^
Luteolin	Cont.	0.19 ± 0.01	168 ± 83	51.3 ± 3.3
	WP	<LOD	-	-
	EPS	0.19 ± 0.01	168 ± 83	51.3 ± 3.3
	WP + EPS	0.05 ± 0.01 ^▲^	24 ± 0	25.8 ± 0.1 ^▲^
Flavan-3-ols				
(+)-Catechin	Cont.	0.13 ± 0.00	168 ± 37	84.1 ± 0.1
	WP	0.13 ± 0.00	168 ± 118	86.3 ± 0.9
	EPS	0.14 ± 0.00	672 ± 290	90.5 ± 0.0
	WP + EPS	0.13 ± 0.00	504 ± 256	88.4 ± 16.5
(−)-Epicatechin-3-gallate	Cont.	0.70 ± 0.00	336 ± 193	169.2 ± 2.4
	WP	0.99 ± 0.00	168 ± 0	328.3 ± 0.0
	EPS	1.40 ± 0.00 *	336 ± 0	265.9 ± 0.0 *
	WP + EPS	4.56 ± 0.26 ^▲^	168 ± 0	783.4 ± 328.0 ^▲^
(−)-Epigallocatechin-3-gallate (EGCG)	Cont.	1.07 ± 0.25	168 ± 193	400.0 ± 61.5
	WP	1.07 ± 0.00	168 ± 33	406.8 ± 0.1
	EPS	0.88 ± 0.00 *	336 ± 0	256.6 ± 0.0
	WP + EPS	2.18 ± 0.12 ^▲^	168 ± 0	596.3 ± 0.1 ^▲^
Phenolic acids				
3-*O*-Feruloylquinic acid	Cont.	0.21 ± 0.03	504 ± 290	34.9 ± 9.0
	WP	0.45 ± 0.00	24 ± 0	134.8 ± 5.1
	EPS	0.45 ± 0.09 *	24 ± 2	81.0 ± 4.6 *
	WP + EPS	0.42 ± 0.04	24 ± 0	116.1 ± 21.9
3-*O*-*p*-Coumaroylquinic acid	Cont.	0.04 ± 0.00	24 ± 8	8.3 ± 0.7
	WP	0.03 ± 0.03	168 ± 0	6.5 ± 0.1
	EPS	0.26 ± 0.00 *	168 ± 0	68.5 ± 0.1 *
	WP + EPS	0.16 ± 0.01	336 ± 0	65.4 ± 8.3
4-*O*-Caffeoylquinic acid	Cont.	1.42 ± 0.01	504 ± 96	710.6 ± 0.5
	WP	1.61 ± 0.00	504 ± 0	1005.8 ± 0.0
	EPS	1.59 ± 0.00	672 ± 0	963.9 ± 1.7
	WP + EPS	1.43 ± 0.08	672 ± 0	890.8 ± 0.1
4-*O*-*p*-Coumaroylquinic acid	Cont.	0.54 ± 0.14	24 ± 1	317.5 ± 2.8
	WP	0.96 ± 0.00	168 ± 0	606.2 ± 5.5
	EPS	0.54 ± 0.01	24 ± 1	317.5 ± 2.8
	WP + EPS	0.96 ± 0.06	168 ± 0	610.1 ± 0.8
Caffeic acid	Cont.	0.24 ± 0.01	504 ± 0	54.5 ± 0.2
	WP	0.05 ± 0.00	168 ± 0	10.6 ± 0
	EPS	1.79 ± 0.15 *	504 ± 227	479.4 ± 39.1 *
	WP + EPS	0.17 ± 0.01 ^▲^	504 ± 0	78.8 ± 0.0 ^▲^
Ferullic acid	Cont.	0.77 ± 0.02	336 ± 83	494.6 ± 0.4
	WP	0.17 ± 0.00	336 ± 48	98.4 ± 0.1
	EPS	1.64 ± 0.02	672 ± 290	835.8 ± 3.6
	WP + EPS	1.38 ± 0.09	24 ± 8	760.9 ± 0.1
Gallic acid	Cont.	0.58 ± 0.14	672 ± 0	76.9 ± 8.8
	WP	0.20 ± 0.00	24 ± 0	2.5 ± 0.0
	EPS	1.31 ± 0.19 *	672 ± 387	110.1 ± 16.2
	WP + EPS	1.11 ± 0.00	336 ± 0	506.0 ± 0.0 ^▲^
*p*-Coumaric acid	Cont.	<LOD	-	-
	WP	<LOD	-	-
	EPS	0.17 ± 0.03	168 ± 83	38.4 ± 0.0 *
	WP + EPS	0.07 ± 0.00 ^▲^	336 ± 0	27.3 ± 0.3

^a^ The mice were treated intragastrically with blackthorn flower water extract (EPS) at a dose of 25 mg/kg of body weight per day and whey proteins (WP) at a dose of 700 mg/kg of body weight per day. There were six animals per group (*n* = 6). * Statistically significant difference compared to the control group (*p* ≤ 0.05); ^▲^ statistically significant difference compared to the EPS group (*p* ≤ 0.05). The results are presented as the mean ± standard deviation. Abbreviations: Cont.—control; EPS—blackthorn flower water extract; WP—whey proteins; *c*_max_—maximal concentration of the compound in tissue; *t*_max_—average time when the compound reaches *c*_max_; AUC_last_—area under the curve; <LOD—below the limit of detection.

**Table 3:** UPLC analysis and basic pharmacokinetic parameters of detected polyphenols in the kidneys of groups treated with blackthorn flower extract in combination with a protein-enriched diet daily for 28 days.

^a^ Kidney’s Bioavailability		1–28 Day, Subchronic Dose *		
Compound	Group	*c*_max_ (µg/g Tissue)	*t*_max_ (h)	AUC_last_ (h·µg/g Tissue)
Flavonols				
Kaempferol-pentoside	Cont.	2.77 ± 0.02	24 ± 0	1317.0 ± 14.7
	WP	1.33 ± 0.00	24 ± 0	536.7 ± 3.2
	EPS	2.94 ± 0.03	24 ± 0	1381.1 ± 12.5
	WP + EPS	2.76 ± 0.00	24 ± 0	1305.6 ± 8.9
Quercetin-3-*O*-glucoside	Cont.	0.15 ± 0.00	168 ± 83	85.1 ± 1.0
	WP	<LOD	24 ± 0	0.0 ± 0.0
	EPS	0.20 ± 0.00	24 ± 0	121.1 ± 0.6
	WP + EPS	0.14 ± 0.03	672 ± 194	33.4 ± 3.7 ^▲^
Quercetin-3-*O*-rutinoside	Cont.	0.65 ± 0.35	168 ± 19	240.1 ± 100.8
	WP	0.28 ± 0.01	336 ± 0	65.8 ± 15.2
	EPS	2.96 ± 0.02 *	336 ± 0	1764.0 ± 6.7 *
	WP + EPS	0.38 ± 0.00 ^▲^	504 ± 0	89.9 ± 34.3 ^▲^
Quercetin-pentosyl-hexoside	Cont.	0.13 ± 0.00	24 ± 0	86.8 ± 1.2
	WP	0.14 ± 0.00	24 ± 0	91.6 ± 0.3
	EPS	0.41 ± 0.00*	24 ± 8	193.2 ± 1.1*
	WP + EPS	0.36 ± 0.00	24 ± 9	163.9 ± 1.4
Quercetin-rhamnoside	Cont.	1.35 ± 0.02	24 ± 0	609.0 ± 12.7
	WP	0.66 ± 0.00	24 ± 0	395.7 ± 1.5
	EPS	1.46 ± 0.00	24 ± 0	699.5 ± 1.0
	WP + EPS	0.24 ± 0.14 ^▲^	672 ± 388	52.3 ± 11.7 ^▲^
Flavones				
Apigenin	Cont.	0.45 ± 0.01	24 ± 0	128.2 ± 1.0
	WP	0.46 ± 0.00	24 ± 0	134.9 ± 1.2
	EPS	0.96 ± 0.02 *	504 ± 0	349.2 ± 0.6 *
	WP + EPS	0.34 ± 0.00 ^▲^	168 ± 0	119.5 ± 0.0 ^▲^
Luteolin	Cont.	0.13 ± 0.15	672 ± 0	11.3 ± 3.5
	WP	<LOD	-	-
	EPS	0.52 ± 0.19 *	24 ± 0	131.2 ± 13.7 *
	WP + EPS	0.11 ± 0.06 ^▲^	672 ± 388	9.5 ± 5.5 ^▲^
Flavan-3-ols				
(+)-Catechin	Cont.	0.21 ± 0.00	24 ± 0	92.9 ± 1.2
	WP	1.41 ± 0.00	24 ± 0	654.0 ± 3.2
	EPS	1.41 ± 0.01 *	168 ± 0	776.2 ± 1.4 *
	WP + EPS	1.42 ± 0.03	24 ± 3	634.8 ± 95.4
(−)-Epicatechin	Cont.	0.03 ± 0.00	24 ± 0	0.3 ± 0.0
	WP	1.32 ± 0.02	336 ± 38	668.7 ± 2.1
	EPS	1.55 ± 0.00 *	168 ± 0	848.4 ± 0.4 *
	WP + EPS	1.65 ± 0.00	672 ± 0	686.0 ± 1.2
(−)-Epigallocatechin-3-gallate (EGCG)	Cont.	0.83 ± 0.00	24 ± 0	514.9 ± 2.3
	WP	0.91 ± 0.55	168 ± 0	500.7 ± 138.4
	EPS	0.91 ± 0.00	24 ± 0	589.1 ± 2.6
	WP + EPS	0.94 ± 0.14	168 ± 29	592.8 ± 72.6
Phenolic acids				
4-*O*-*p*-Coumaroylquinic acid	Cont.	0.65 ± 0.003	672 ± 291	412.3 ± 11.1
	WP	1.30 ± 0.01	336 ± 0	341.6 ± 25.9
	EPS	2.07 ± 0.04 *	672 ± 0	1143.1 ± 2.6 *
	WP + EPS	0.79 ± 0.01 ^▲^	336 ± 37	272.5 ± 38.0 ^▲^
Ferullic acid	Cont.	0.84 ± 0.56	504 ± 277	261.6 ± 30.79
	WP	0.49 ± 0.20	504 ± 246	116.7 ± 27.8
	EPS	5.45 ± 0.00 *	504 ± 0	1354.5 ± 1.5 *
	WP + EPS	1.05 ± 0.00 ^▲^	504 ± 0	172.0 ± 0.5 ^▲^

^a^ The mice were treated intragastrically with blackthorn flower water extract (EPS) at a dose of 25 mg/kg of body weight per day and whey proteins (WP) at a dose of 700 mg/kg of body weight per day. There were six animals per group (*n* = 6). * Statistically significant difference compared to the control group (*p* ≤ 0.05); ^▲^ statistically significant difference compared to the EPS group (*p* ≤ 0.05). The results are presented as the mean ± standard deviation. Abbreviations: Cont.—control; EPS—blackthorn flower water extract; WP—whey proteins; *c*_max_—maximal concentration of the compound in tissue; *t*_max_—average time when the compound reaches *c*_max_; AUC_last_—area under the curve; <LOD—below the limit of detection.

**Table 4:** The effects of high-protein diet combined with daily consumption of *P*. *spinosa* flower extract (over 28 days) on the representative markers of tissue antioxidative defense system (superoxide dismutase, SOD; catalase, CAT; total glutathione, GSH) in the intestine, liver, and kidneys of C57BL/6 mice, assessed on the 28th day of experiment.

Groups ^a^	MDA (nmol/mg Proteins)	CAT (U/mg Proteins)	SOD (U/mg Proteins)	GSH (mU/mg Proteins)
	Small Intestine			
Cont.	1.52 ± 0.24	10.47 ± 2.05	10.17 ± 4.31	76.79 ± 12.69
WP	1.02 ± 0.28	10.96 ± 1.42	25.37 ± 3.31 #	70.01 ± 8.53
EPS	0.52 ± 0.09 *	8.78 ± 1.50	12.53 ± 2.17 *	119.95 ± 12.90 *
WP + EPS	0.98 ± 0.20	7.87 ± 1.13	17.11 ± 3.35 ^▲^	63.43 ± 20.71 ^▲^
	Liver			
Cont.	1.69 ± 0.41	93.91 ± 35.84	15.44 ± 1,49	109.81 ± 25.45
WP	1.58 ± 0.50	145.35 ± 42.15	35.04 ± 5.04	157.48 ± 22.47
EPS	0.91 ± 0.22 *	107.94 ± 25.75 *	24.58 ± 6.16 *	138.15 ± 26.51 *
WP + EPS	1.26 ± 0.39	175.20 ± 52.56 ^▲^	27.42 ± 5.54 ^▲^	167.71 ± 29.55 ^▲^
	Kidney			
Cont.	1.75 ± 0.38	60.92 ± 14.41	12.78 ± 1.81	62.64 ± 9.23
WP	1.26 ± 0.42	42.12 ± 9.48	26.28 ± 2.67	79.34 ± 12.75
EPS	1.06 ± 0.23 *	80.9 ± 14.78 *	22.16 ± 3.13 *	106.10 ± 16.92 *
WP + EPS	1.17 ± 0.35	61.66 ± 9.25 ^▲^	18.29 ± 2.55 ^▲^	73.96 ± 17.09

^a^ The mice were treated intragastrically with blackthorn flower water extract (EPS) at a dose of 25 mg/kg of body weight per day and whey proteins (WP) at a dose of 700 mg/kg of body weight per day. There were six animals per group (*n* = 6). * Statistically significant difference compared to the control group (*p* ≤ 0.05); ^▲^ Statistically significant difference compared to the EPS group (*p* ≤ 0.05); # Statistically significant difference compared to the WP + EPS group. The results are presented as the mean ± standard deviation. Abbreviations: Cont.—control; EPS—blackthorn flower water extract; WP—whey proteins.

## Data Availability

The original contributions generated for this study are included in the article; further inquiries can be directed to the corresponding author.
